# Two step approach for detecting and segmenting the second mesiobuccal canal of maxillary first molars on cone beam computed tomography (CBCT) images via artificial intelligence

**DOI:** 10.1186/s12903-025-06796-4

**Published:** 2025-09-08

**Authors:** Sally Mansour, Enas Anter, Ali Khater Mohamed, Mushira M. Dahaba, Arwa Mousa

**Affiliations:** 1 Oral and Maxillofacial Radiology Department, Cairo university, Cairo, Egypt; 2 Department of Computer Science, Faculty of Computer Science, October University for Modern Sciences and Arts (MSA), Giza, Egypt

**Keywords:** Artificial intelligence, CNN, Deep learning, Second mesiobuccal canal, MB2 canal, Endodontics, Cone beam computed tomography, CBCT

## Abstract

**Aim:**

The purpose of this study was to assess the accuracy of a customized deep learning model based on CNN and U-Net for detecting and segmenting the second mesiobuccal canal (MB2) of maxillary first molar teeth on cone beam computed tomography (CBCT) scans.

**Methodology:**

CBCT scans of 37 patients were imported into 3D slicer software to crop and segment the canals of the mesiobuccal (MB) root of the maxillary first molar. The annotated data were divided into two groups: 80% for training and validation and 20% for testing. The data were used to train the AI model in 2 separate steps: a classification model based on a customized CNN and a segmentation model based on U-Net. A confusion matrix and receiver-operating characteristic (ROC) analysis were used in the statistical evaluation of the results of the classification model, whereas the Dice-coefficient (DCE) was used to express the segmentation accuracy.

**Results:**

F1 score, testing accuracy, recall and precision values were 0.93, 0.87, 1.0 and 0.87 respectively, for the cropped images of MB root of maxillary 1st molar teeth in the testing group. The testing loss was 0.4, and the area under the curve (AUC) value was 0.57. The segmentation accuracy results were satisfactory, where the DCE of training was 0.85 and DCE of testing was 0.79.

**Conclusion:**

MB2 in the maxillary first molar can be precisely detected and segmented via the developed AI algorithm in CBCT images.

**Trial registration:**

Current Controlled Trial Number NCT05340140. April 22, 2022.

## Introduction

Every endodontist aims to achieve a successful nonsurgical root canal treatment. Before beginning the endodontic procedure, the dentist must first recognize and thoroughly understand root canal anatomy and the various root morphologies. The morphology of the mesiobuccal (MB) root of maxillary molars has attracted the attention of many researchers in recent decades [1]. This is because this root frequently has two major root canals, known as the 1st mesiobuccal canal (MB1) and the 2nd mesiobuccal canal (MB2), as well as a high frequency of fine anatomical structures such as loops, auxiliary canals, intercanal communications, and apical ramifications [2], resulting in a very complex canal system. The orifice of MB2 is usually located 2 mm mesially from MB1 and 3.5 mm palatally in the sub-pulpal groove [3], and is frequently hidden behind the pulp chamber wall shelf [4].

Unfortunately, MB2 canal often goes undetected in routine clinical practice due to the difficulty of identifying it without magnification or specialized lighting, and thus it is left untreated [5]. This explains why a recognizable percentage of root canal therapies for maxillary molars are unsuccessful [6–8]. It was found that the majority of missed canals requiring endodontic retreatment are found in the maxillary first molar [9, 10] with 93% of these cases specifically located in the mesiobuccal root [11].

In the literature, the presence of MB2 canal in the maxillary molars ranges from 10-95%; this wide range depends on multiple factors, such as the method of MB2 detection either clinical or radiographic [2, 10, 12], and ethnic and demographic factors such as location, age and sex, related to the studied population [13, 14]. A recent systematic review, included 22 studies and 41 distinct population groups, investigated the prevalence of MB2 canal in maxillary first molars, revealing a combined prevalence rate of 69.6% [15].

In accordance with a joint position statement of the American Association of Endodontics and American Academy of Oral and Maxillofacial Radiology [16] and, more recently, an updated consensus of an expert committee in conjunction with the European Society of Endodontology [17], regarding the use of cone beam computed tomography (CBCT) in clinics, small field of view (FOV) CBCT is recommended when complex anatomy is expected to exist. Therefore, CBCT is currently considered the gold-standard imaging technique for evaluating the presence of MB2 canal in the clinic [14, 18, 19].

One of the advanced applications of CBCT in endodontics is the generation of a 3D virtual representation of the pulp space via segmentation [20]. The creation of 3D model of pulp space morphology in endodontics can be seen as an asset for diagnosis, treatment planning, patient communication, and preclinical training. This is because it provides realistic reproduction of the pulp, which greatly improves the understanding of the root canal system [21]. The process of segmenting the pulp space, specifically the root canal, is very challenging because of its thin and narrow path.

The traditional methods for pulp segmentation on CBCT images, including manual and semiautomated approaches, have their own limitations. Manual segmentation methods require advaned level of experience and are highly time consuming [22], whereas semiautomated approaches lack standardized voxel values in software programs for the segmentation of pulp space [23, 24].

Technological advances in oral and maxillofacial radiology have extended beyond imaging modalities and hardware. The quality and depth of radiology’s contribution to patient care and population health, as well as radiologists’ workflows, are expected to undergo a major revolution in the next ten years with the application of artificial intelligence (AI).

Deep learning (DL) is considered a subdivision of AI that uses several hidden layers of neural networks to simulate how biological neurons behave and gradually “learn” by taking attributes from the input data [25]. Neural networks, such as convolutional neural networks (CNNs), constitute the core of most of these implementations [26, 27].

Indeed, the challenges associated with segmenting dento-maxillofacial features have been shown to be manageable using DL [28–30]. In a recent systematic review evaluating the performance of AI for pulp space segmentation, most of the included studies demonstrated a high degree of accuracy in their respective segmentation methods. However, the results were variable among different structures, where the automated pulp chamber segmentation was more accurate than that of the root canal segmentation [31, 32], and the segmentation of single-rooted teeth were more accurate than the multirooted teeth [21, 33].

Although several recent studies have demonstrated the feasibility of identifying canals via AI [33–35], 2 studies only have explored the detection of the correct number and anatomy of canals in the MB root in maxillary molars [36]; [37]. The aim of the present study was to develop a new approach using deep learning model based on CNN to accurately classify MB root canals without the need for prior segmentation, followed by a U-Net-based model to perform automated segmentation of the MB root canals in the maxillary first molar teeth on CBCT images.

## Methods

### Study design

Our study is a retrospective study in which the data collection was considered before the performance of index tests and reference standards. It is considered a diagnostic accuracy study because the accuracy of a newly developed deep learning model (DLM) in the automatic detection and segmentation of the MB2 canal in maxillary first molars is assessed by comparison with the opinion of the experienced radiologists, which represents the ground truth and its results are represented in the form of accuracy, sensitivity (recall), positive predictive value (precision) and receiver operating curve. In accordance with the code of ethics, this study was undertaken after the approval of the Research Ethics Committee of the Faculty of Dentistry, Cairo University. on 25/1/2022, and it complies with the Declaration of Helsinki (2013).

Patient declaration of consent was obtained via a Helsinki declaration consent form in their native language (Arabic). Provided is the English version of said form and a signed Arabic one by one of the study participant.

### Sample size calculation

A power analysis was conducted to ensure sufficient power for a two-sided statistical test of the null hypothesis that the accuracy of the DL model is equivalent to that of the radiologist’s opinion. A 95% confidence interval and a specificity value of 88.0% were used for the DL group, as derived from a prior study [38], alongside a 100% specificity for the ground truth.

The sample size calculation was approved by the Medical Biostatistics Unit, Faculty of Dentistry, Cairo University, on 17/1/2022.

### Radiographic dataset

The CBCT scans used in this study were obtained from the CBCT database available at the department of Oral and Maxillofacial Radiology, Faculty of Dentistry, Cairo University, Cairo, Egypt. The scans were obtained as a part of the diagnosis and treatment planning of the included patients.

All the selected scans were acquired using a voxel size of no more than 0.075 mm and featuring fully erupted maxillary first molars with complete root formation. Exclusions comprised maxillary first molars exhibiting developmental anomalies, external or internal root resorption, root canal calcification, prior root canal treatment, post-restoration, or root caries. Additionally, CBCT images of suboptimal quality, those with significant scattered artifacts hindering accurate assessment, as well as scans with a field of view (FOV) extending beyond a single arch or employing a voxel size exceeding 0.075 mm, were also excluded.

All the included scans (37 scans) were taken via a Planmeca Promax 3D MID CBCT machine, and the imaging protocol was standardized as follows: exposure parameters: 87 KVp, 8 mA, exposure time 12 s; voxel size 0.075 mm; and FOV 5 × 5-5 × 8 cm. Thirty-seven maxillary molars with MB2 canals (*n* = 20) and without MB2 canals (*n* = 17) were included.

### Radiographic annotation (ground truth)

CBCT scans were imported into 3D slicer software program (*open-source free software version 5.2.2*,* Harvard University*,* USA*) for data annotation, which was performed in 2 separate steps: (classification and segmentation).

**Fig. 1:**
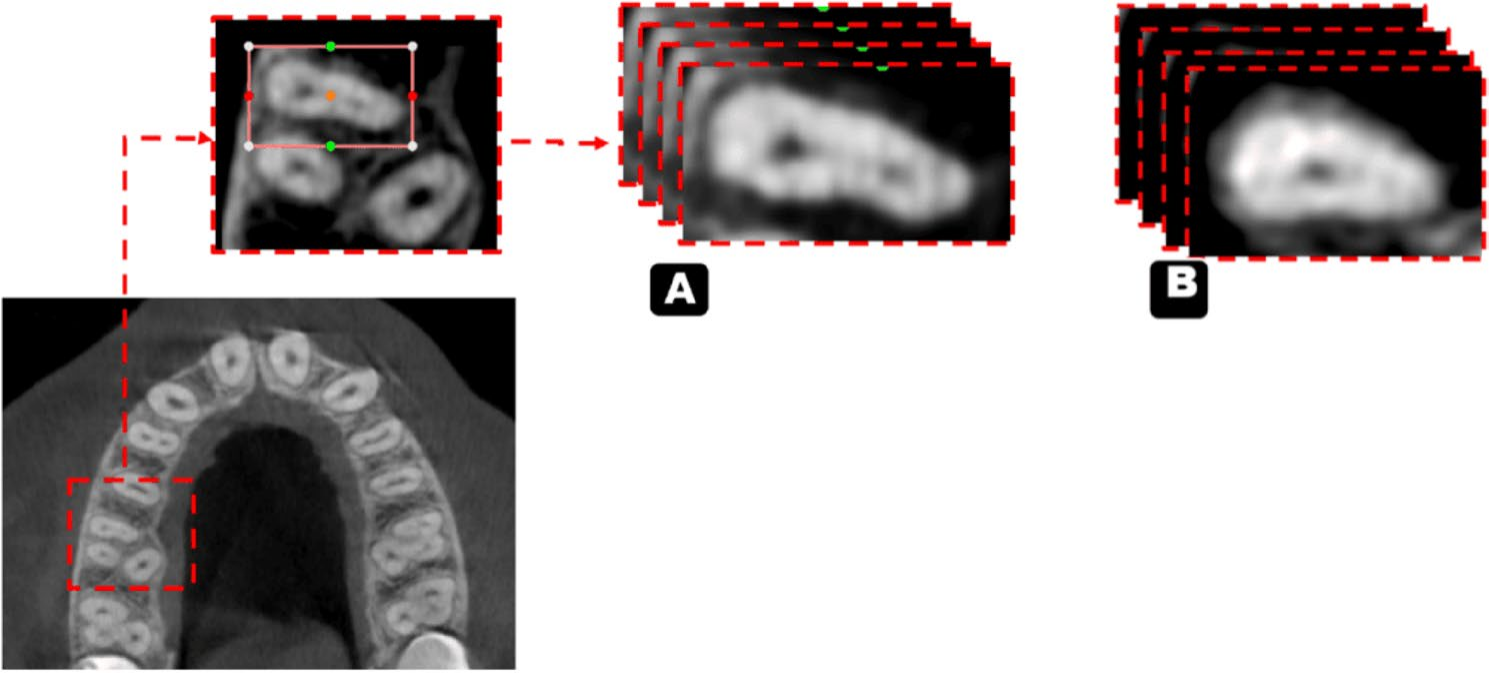
Illustration of axial images used for training the classification model a. With MB2 B. Without MB2

Regarding the classification stage, radiologist-dependent MB2 canal detection was performed on axial cuts along the entire length of the root and then classified into the presence or absence of the MB2 canal, which was confirmed and agreed upon by 2 oral and maxillofacial radiologists (OMFRs) (8 & 15 years of experience) (Fig. 1). This classification serves as the ground truth for the first process.

**Fig. 2:**
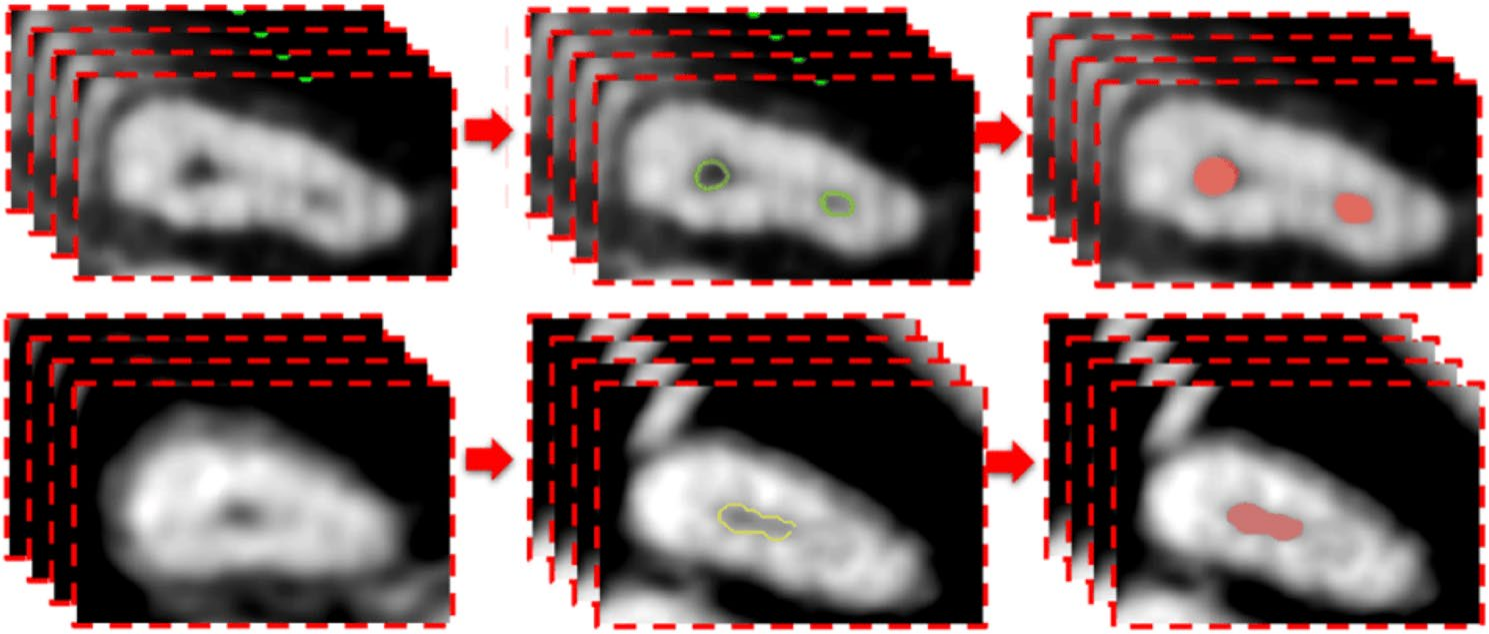
Illustration of axial images used for training the segmentation model (with and without MB2)

In the segmentation stage, the cropped CBCT scans were segmented via the same software (3d slicer, version 5.2.2) through the segment editor module. This module edits overlapping segments, displays them in both 2D and 3D views, creates segmentation by interpolation of few slices, and allow editing in any orientation. The process was performed on the axial slices and started with a semiautomatic segmentation tool called “level tracing”, which allows for automatically selecting the range of thresholds for the root canal label (Fig. 2). This process was performed every 5 slices, and segmentation was completed through an interpolation tool. After segmentation of all slices was initially completed, each sagittal, coronal, and axial slice was reviewed and manually revised and agreed upon the 2 experienced radiologists.

Finally, all images were saved in the NIfTI format, with the segmentation images stored as binary label maps. This format was chosen as it is the most widely used representation due to its ease of editing. Subsequently, all files were shared with a computer science expert for further analysis.

### Development of the AI models

The classification and segmentation algorithms were developed in the Python environment *(v3.9.19; Python Software Foundation*,* Wilmington*,* DE*,* USA)* utilizing the TensorFlow library. Mathematical processing in the model’s training was performed with a Lenovo Legion y540, Intel i7-9750 H, 16GB DDR4 RAM, and GTX 1660ti 6GB *(Lenovo Group Limited China)* at the Faculty of Computer Science MSA University, Cairo, Egypt.

To improve the visual quality of the axial slices, image enhancement techniques such as density normalization were applied, and data augmentation was performed in a layer during the model training as follows: random horizontal and/or vertical flips were performed, followed by a random rotation with an angle from − 30 to 30.

### Data split

A total of 37 anonymized CBCT volumes with DICOM files were converted to the NIfTI file format. The dataset was randomly separated into (training and validation (80%) and testing (20%)) groups.

Our study approached the process of detection and segmentation of MB2 canals separately through two sequential models; a convolutional neural network (CNN) based model for classification process and a U-Net-based model for segmentation process.

### Classification model

The classification model was based on a CNN because CNNs are highly effective at handling image data because of their ability to automatically learn spatial hierarchies of features through convolutional layers. Unlike traditional machine learning models, which rely on manual feature extraction, CNNs can capture complex patterns such as edges, textures, and shapes directly from raw images. This makes them particularly suitable for image classification tasks. The convolutional layers scan the input images via filters, detecting essential features at various levels of abstraction, whereas the pooling layers reduce dimensionality, improving computational efficiency and minimizing overfitting. In addition, CNNs are invariant to transformations such as scaling, shifting, and rotation, which improves the robustness of the model when classifying diverse or distorted images. The ability of CNNs to generalize well across large datasets and their effectiveness in automating feature extraction make CNNs the optimal choice for building powerful, scalable image classification models.

### CNN model architecture

**Fig. 3:**
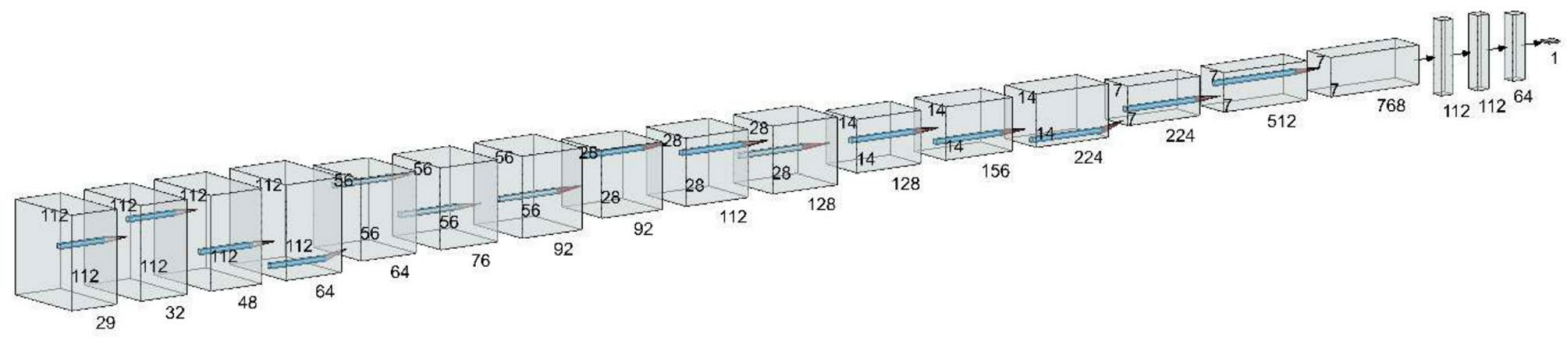
Pipeline for the CNN model architecture used for MB2 classification

The model starts by reading the 37 Nii images into sequential pngs; then, each scan is sampled into 29 channels only to capture the most relevant slices of the volumetric data, with a focus on regions that contain significant diagnostic information. This sampling process was designed to reduce the complexity of the 3D Nii images while preserving enough depth to ensure that the model could effectively distinguish between different features across the slices. By selecting 29 channels, the model maintains a balance between computational efficiency and the retention of crucial spatial information from the original data. These channels were treated as separate input layers, allowing the CNN to process the images as a sequence of related 2D slices that collectively represent the 3D structure. After that, the images were resized to 128 × 128 to standardize their dimensions, facilitating batch processing and improving computational performance during training. This approach ensures that the input data are of consistent size while maintaining the integrity of the key image features needed for accurate classification and also ensures uniform input dimensions for the CNN model. This resizing step also helps reduce the computational load while maintaining the essential features necessary for classification. Following resizing, each image was normalized to ensure that the pixel values were in a standardized range, improving convergence during training by reducing the risk of vanishing or exploding gradients. The 29 channels of each scan, which represent different slices or layers, were stacked and treated as distinct inputs to capture the depth and context of the 3D structure present in the original Nii images. These channels allowed the model to learn spatial correlations across multiple layers, enhancing its ability to identify subtle patterns. After preprocessing, the data were split into training, validation, and test sets to ensure that the model could generalize well to unseen data. This entire pipeline helps optimize the model’s performance by balancing computational efficiency with the preservation of critical spatial and visual information within medical scans (Fig. 3).

**Fig. 4:**
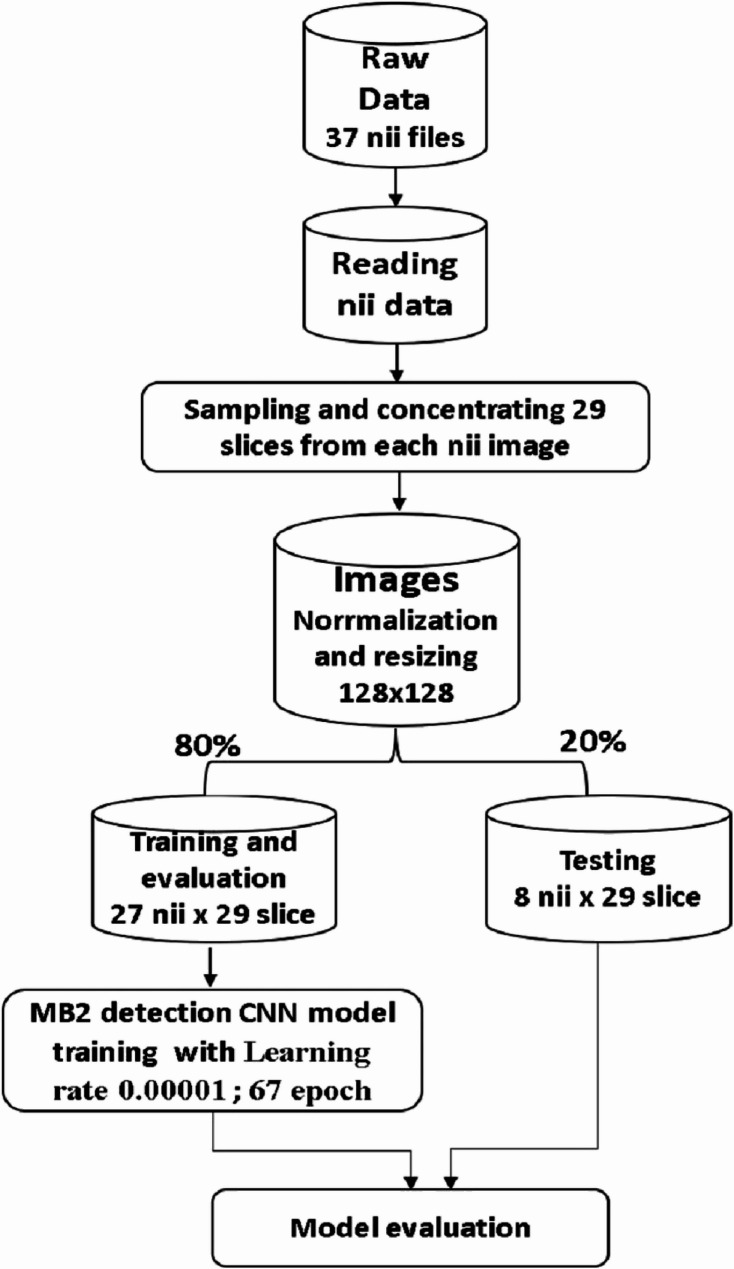
Diagram of the MB2 classification model development steps

The training dataset was used to train the DL model, and the validation dataset was used for early stopping criteria. The classification model underwent training for 67 epochs with a learning rate of 0.00001, the patch size was 8, and the optimizer used was Adam, with a total of 2,005,125 parameters extracted. After that, the customized deep learning model was tested on an independent test dataset, and the best model was recorded (Fig. 4).

### Segmentation model

The segmentation model was based on U-Net because of its proven effectiveness in biomedical image segmentation tasks, especially when dealing with limited data. U-Net’s architecture is particularly well-suited for capturing fine-grained details in images, because of its symmetric encoder-decoder structure.

The encoder path allows the model to capture contextual information through downsampling, whereas the decoder path uses upsampling to restore spatial resolution, ensuring precise localization. Additionally, the skip connections between the corresponding layers in the encoder and decoder allow the model to retain critical high-resolution features, which are essential for accurate segmentation of anatomical structures.

**Fig. 5:**
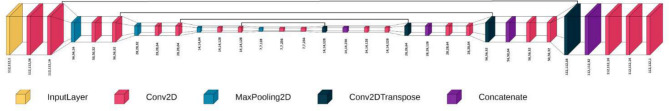
Pipeline for the U-Net model architecture used for MB2 segmentation

**Fig. 6:**
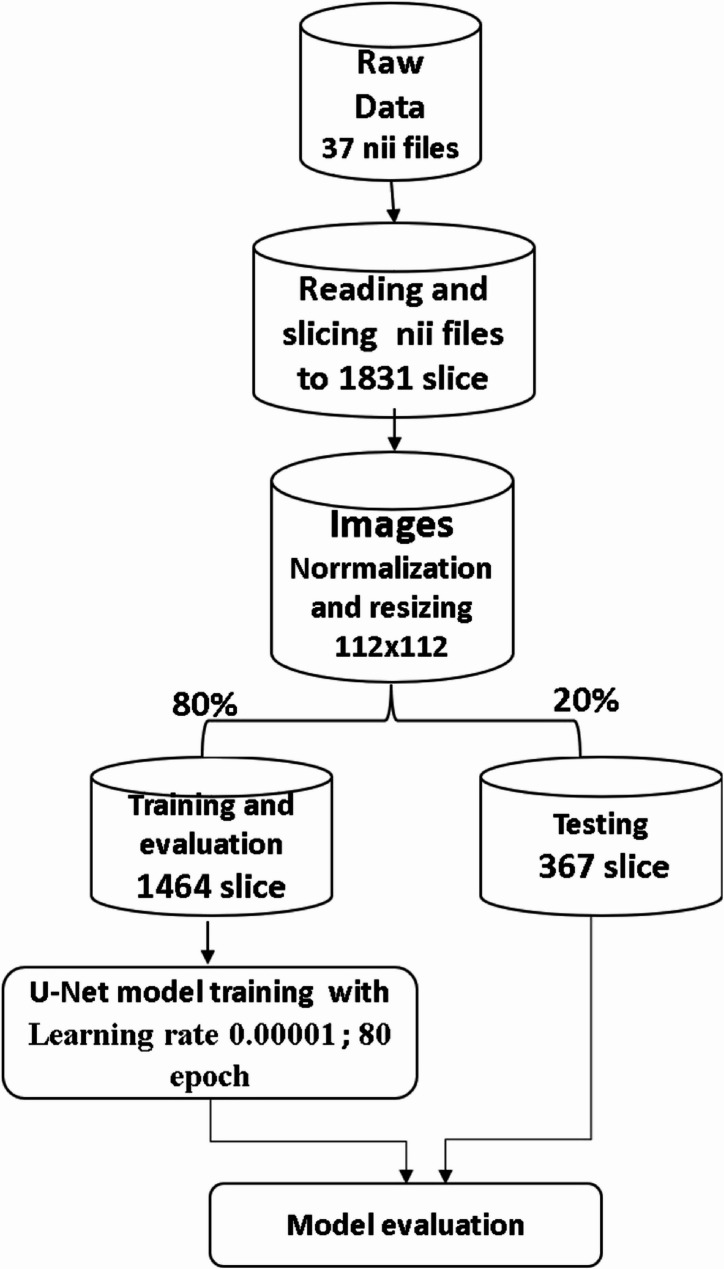
Diagram of the MB2 segmentation model development steps

This architecture helps balance the need for both a global context and detailed localization, making U-Net highly effective for medical imaging tasks where precision is paramount. By leveraging U-Net, the model can effectively learn to identify and segment regions of interest from the 1831 axial slices with relatively few training examples, making it a powerful tool for medical image segmentation. The model started by reading the 37 Nii images into 1831 png axial slices. After that, the images were resized to 112 × 112 before the data were split into 1464 slice for training and 367 slice for testing (Fig. 5). The model started training for 80 epochs with a learning rate of 0.00001, the patch size was 16, and the optimizer used was Adam, with a total of 1,940,817 parameters extracted (Fig. 6).

#### Evaluation

The accuracy of the deep learning model (DLM) versus the ground truth (GT) was evaluated via the following 2 methods:

1. Canal detection accuracy using the classifier DLM:

A dichotomized outcome of “presence of MB2” and “absence of MB2” per MB root of DLM was compared with that of GT, where the GT was determined and agreed upon by 2 maxillofacial radiologists with 8 and 15 years of experience.

The results of the test group were put into a confusion matrix of true positive (TP), false positive (FP) and false negative (FN) parameters, where (TP) is the accurate prediction of the image with MB2, (FP) is the incorrect prediction of the image with MB2 and (FN): is the incorrect prediction of the image without MB2, and (TN) is the correct prediction of the image without MB2.

On the basis of this confusion matrix, the precision, recall (sensitivity) and F1 score were calculated and graded according to the ranking for diagnostic tests by Leonardi Dutra et al. [39], with scores of 80% considered excellent outcomes, scores between 70% and 80% good, scores between 60% and 69% fair, and scores of 60% poor. Roots with 1 canal were used as the control group.

- The Accuracy, evaluates the correct predictions generated by the model throughout the complete dataset. The calculation is the ratio of true positives (TPs) and true negatives (TNs) to the total number of samples.
- The precision, or positive predictive value, evaluates only true positives among all positive predictions generated by the model. It is determined by the ratio of true positives (TPs) to the total number of true positives and false positives (FPs).
- Recall, also referred to as the sensitivity or true positive rate, quantifies the ratio of true positive forecasts to all real positive cases, thereby accounting for missed positives. It is determined by the ratio of true positives (TPs) to the total number of true positives and false negatives (FNs).
- F1 score— The F1 score is a statistic that equilibrates precision and recall. It is computed as the harmonic mean of precision and recall.

Accuracy assesses the overall correctness of the model’s predictions, whereas precision and recall concentrate on the quality of positive and negative predictions, respectively. The F1 score offers an appropriate ratio between precision and recall, rendering it a more balanced indicator for assessing classification models. The area under the curve (AUC) of the receiver operating characteristic (ROC) curve was also calculated.

2. The segmentation accuracy was measured through voxel matching via the Dice similarity coefficient calculated for the root canal label:

The set of pixels labelled as root canals in the segmented image generated by our U-Net-based model was compared with those labelled by the radiologist (GT).

The Dice similarity coefficient, or simply the Dice coefficient, is a statistical tool that measures the similarity between two sets of data. This index has become a common metric in the validation of image segmentation algorithms created with AI.

The equation for this concept is:

$2\ast\left|\mathrm X\cap\mathrm Y\right|/\left(\left|\mathrm X\right|+\left|\mathrm Y\right|\right)$, where X and Y are two sets:

- |X| represents the number of elements in set X (voxels segmented by the DLM), and |Y| represents the number of elements in set Y (voxels segmented by the radiologist (GT)).
- ∩ is used to represent the intersection of two sets and represents the elements that are common to both sets.

## Results

### Classification results

The simple classifier model successfully predicted the presence of Mb2, which was properly represented by the F1-score, accuracy, recall and precision values. The loss function and the AUC values of both training and testing are also measured and are shown in Table 1.

**Table 1:** Results of both training and testing of the classification model

	Training	Testing
F1 score	0.76	0.93
Accuracy	0.76	0.87
Precision	0.73	0.87
Recall	0.78	1.0
Loss	0.52	0.4
AUC	0.8	0.57

**Fig. 7:**
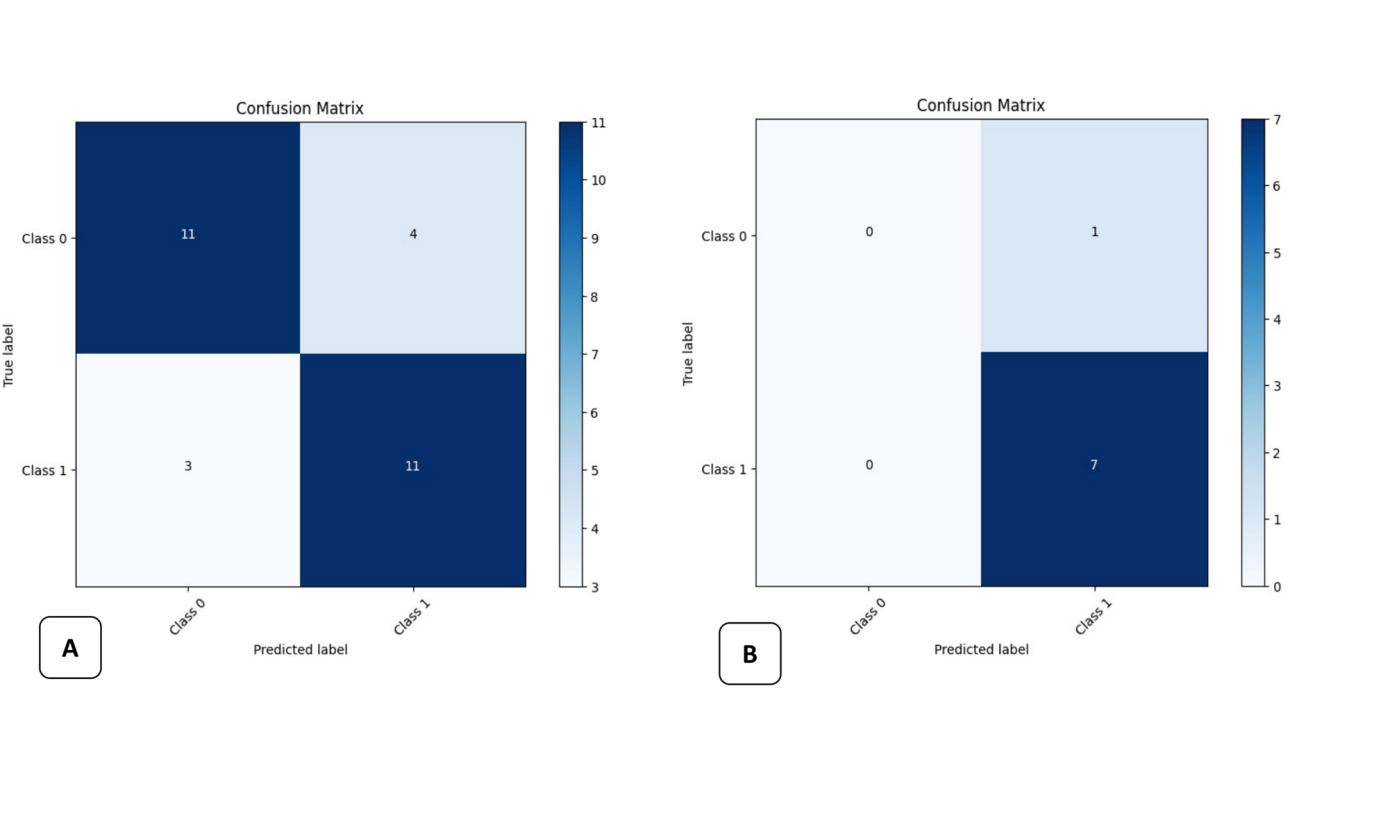
Confusion matrix of the classification model during **A**. training and **B**. testing the images

**Fig. 8:**
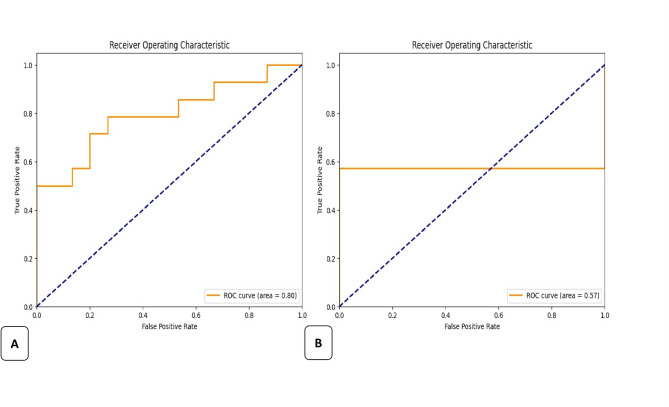
ROC curve of the classification model during **A**. Training and **B**. Testing the images

Figure 7 (a-b) presents the confusion matrix of our classification results during training and testing. Figure 8 (a-b) presents the reciever-operating characteristic (ROC) curves of this group during training and testing.

### Segmentation results

**Fig. 9:**
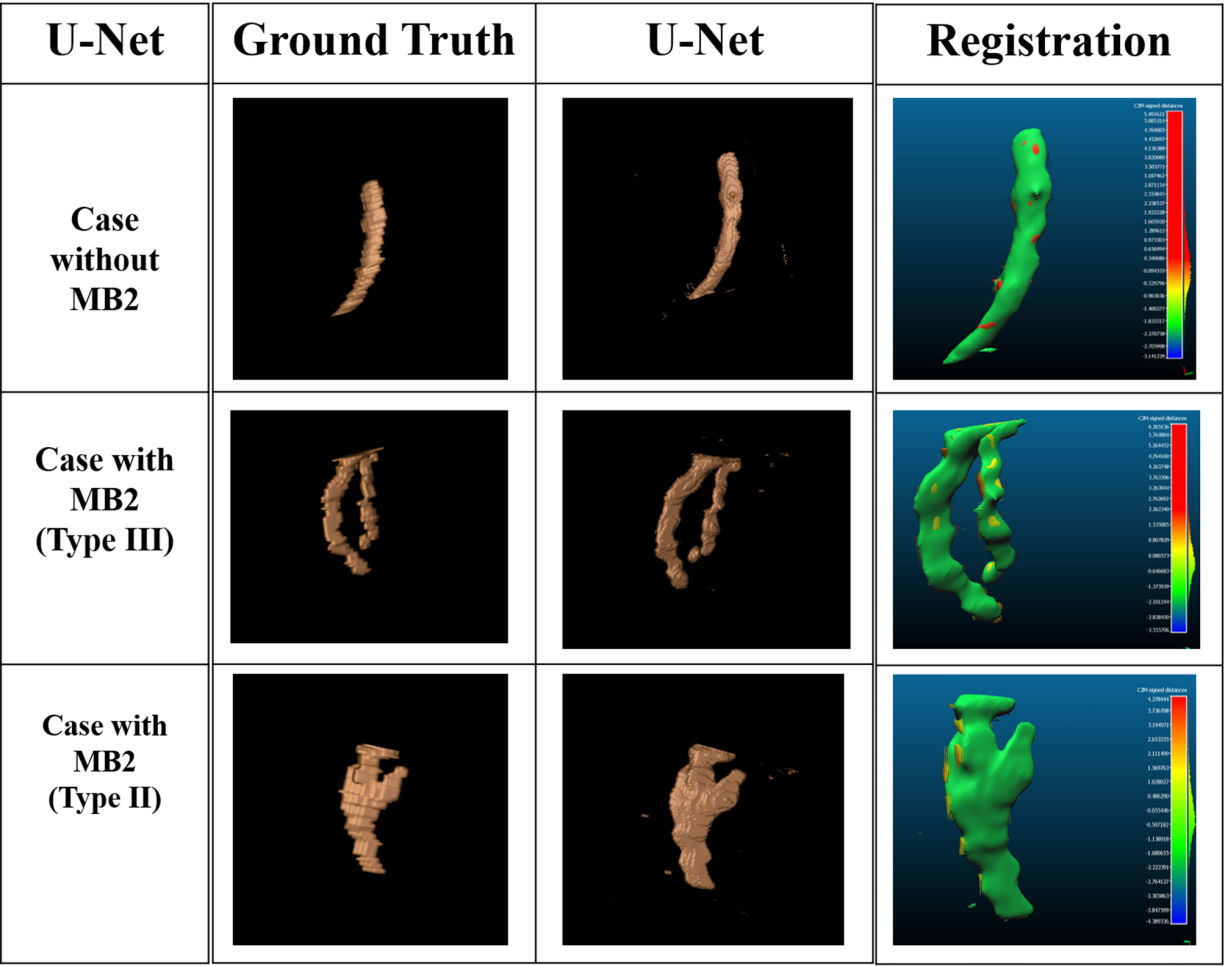
Three-dimensional reconstructed images of the U-Net model performance in comparison with the ground truth

The results of segmentation were expressed in DCEs, where the DCE of training was 0.8548, whereas the DCE of testing was 0.7952. The training loss was binary cross-entropy, where the training loss was 0.0164, and the testing loss was 0.0257 (Fig. 9).

## Discussion

One of the most common causes of endodontic failure is the failure to locate root canals during endodontic treatment. Endodontic retreatment involves treating missed canals, with 93% of all missed canals being on the maxillary first molar and 44% being on maxillary second molar, specifically MB2 [9–11]. From an economic perspective, retreatment is not only costly to the patient but also time-consuming for both the patient and the dentist. Accordingly, finding an automatic tool that can detect the presence or absence of MB2 canal and identify its anatomical variation is highly important. Our study investigated the use of artificial intelligence, particularly customized DLMs, for the automated detection and segmentation of the MB2 canal in maxillary 1 st molars in a localized CBCT FOV.

CBCT is considered the gold-standard imaging technique for evaluating the presence of the MB2 canal [14, 18, 19]. Since the detection accuracy and reliability of MB2 canals increased as the resolution improved [40], a small FOV (50 × 50 mm, 50 × 80 mm) and small voxel sizes (0.075 mm) were included in our study to use high-resolution images to create the AI model.

For image annotation, 3D slicer software *(Harvard University*,* USA*) was used for cropping the images and for the segmentation process. This software has several advantages, including being a free-source program with multiple tools for segmentation, a convenient interface and compatibility with multiple operating systems (Windows, Mac OS X, and Linux) [41]. To achieve more accurate segmentation results using 3D slicer, we used interpolation to refine the voxel size of the original CBCT images, which resulted in making the edges of the segmentation more coherent, in addition to saving time during the process.

Our dataset comprised 37 manually labelled CBCT scans, which were strategically divided into training, validation, and testing sets. This division was guided by the recommendations of a computer science expert to ensure a balanced model, effectively mitigating the risks of overfitting or underfitting. One of the key strengths of the U-Net architecture employed in our study lies in its innovative network and training strategy, which incorporates data augmentation to maximize the efficiency of available annotated samples [42]. This approach allows the model to generalize effectively, even with a relatively small dataset.

While 3D image analysis typically demands extensive computational resources, our method simplifies the process by projecting cropped axial views into 2D slices, reducing spatial complexity while maintaining diagnostic precision. By integrating optimal voxel resolution, refined preprocessing techniques, and an advanced DLM, our approach achieves both high accuracy and clinical applicability.

Although many studies have used CBCT imaging to detect anatomical variations in the root canal system using AI [33–35], only 2 studies in the literature have applied deep learning systems to detect MB2 canal [36, 37]. Albitar et al. utilized U-Net for MB2 canal detection and segmentation in CBCT images, focusing exclusively on identifying missed MB2 canals in endodontically treated maxillary molars, whereas our study focused on detection of MB2 canals in maxillary 1 st molars without prior endodontic treatment, serving as a critical diagnostic step before initiating endodontic procedures.The second publication was for Duman et al. who similarly segmented MB2 canals in axial CBCT slices [37]. However, our work extends beyond segmentation by incorporating a classification-based CNN model, eliminating the need for manual annotation during detection, which significantly reduced the time and effort typically required.

The findings in the current study indicate the following outcomes: initially, the deep learning algorithm exhibited steadily increasing performance in terms of sensitivity (recall) in identifying MB2 canals, increasing from 76% during the training phase to 87% in the testing phase, with an overall precision of 87% and no false negative results for the detection task. Furthermore, when the segmentation performance was assessed using a custom metric, the model attained a Dice coefficient of 0.854 for the training set and 0.795 for the testing set. The results indicate that the CNN model has a high likelihood of generating real positive outcomes and a low likelihood of false positives, indicating a high probability of administering necessary treatment. However, ROC curve analysis revealed a notably low AUC of 0.57 during testing compared to 0.8 during training, indicating that despite high apparent classification metrics, the model’s actual discriminative ability on unseen data is limited.The segmentation results indicate that our U-Net model excels in segmenting the MB2 canal in maxillary first teeth.

With respect to the accuracy of detection of MB2, our results were more accurate than those of Albitar et al., who reported 80% accuracy compared with 87% accuracy in our study. This can be justified by using CBCT scans of endodontically treated teeth in their study, that definitely have radiographic streaks, whereas in our study, scans with any artefacts were excluded, and the architecture of U-Net enhanced the image before detection [36].

In contrast, our results were less accurate than those of Duman et al., who reported that the sensitivity of the MB2 canal segmentation model was 0.92. The greater quantity of data used in addition to the use of a pretrained model may contribute to the better results they obtained [37].

Numerous studies have been conducted to segment the root canal system utilizing deep learning models; the majority have employed two-dimensional (2D) images, such as periapical or panoramic radiographs, whereas a limited number have focused on root morphology using CBCT images [33, 35, 43–45].

Regarding the utilization of 2D images, Jeon et al. studied the identification of C-shaped canals in mandibular second molars on panoramic radiographs via a deep learning algorithm called X-ception [45]. The results of this study revealed that the AUC value of this CNN model (0.982) was significantly greater than those of a radiologist (0.872) and an endodontist (0.885).

In the same context, Hiraiwa et al. compared the diagnostic performance of two different deep learning models (AlexNet & GoogleNet) for classifying the root morphology of mandibular first molars on panoramic radiographs with that of experienced radiologists. Sixty mandibular first molars from scans of 400 patients who had not undergone root canal treatment were used to train and test the models using CBCT images as the gold standard. The radiologists demonstrated an accuracy of 81.2%, sensitivity of 80.2%, and specificity of 82.0%, whereas the deep learning system displayed slightly superior performance metrics, particularly in specificity, with significant differences noted (Mann Whitney U test; AlexNet vs. radiologists, *p* = 0.0445; Google Net vs. radiologists, *p* = 0.0424) [43].

Considering the use of CBCT images for model creation, Sherwood et al. utilized CBCT images to train a deep learning system for the identification of C-shaped canals in mandibular second molars, employing U-Net, residual U-Net, and Xception U-Net architectures [35]. The C-shaped canals in their study were categorized into subgroups during detection, with Xception U-Net and residual U-Net architectures demonstrating superior performance compared with U-Net. In a separate study, the AlexNet and GoogleNet systems were employed for the differential diagnosis of single or multiple roots in the distal roots of 760 mandibular first molars, utilizing panoramic radiographs from 400 individuals. The diagnostic efficacy of these two CNN designs was equivalent to or superior to that of a radiologist with over 20 years of expertise. The mean sensitivity values were 78% for Xception U-Net, 75% for residual U-Net, and 72% for U-Net.

Duan et al.. assessed the precise segmentation of unobturated teeth and pulp cavities employing a two-phase deep learning methodology; the initial phase utilized CBCT reconstructed panoramic radiographs to delineate single and multirooted teeth through a region proposal network (RPN) and feature pyramid network (FPN), unlike our study which used axial slices rather than reconstructed panoramic views [33]. The Dice coefficient values were very high for both single and multirooted teeth, at 95% and 96%, respectively, whereas the values for the pulp cavity were somewhat lower, at 88% for single and 86% for multirooted teeth.

Recently, AI in the field of endodontics has gained much popularity, especially in the context of CNNs and U-Net architecture. Most endodontic related AI studies, which are based on CBCT images, address periapical pathosis detection and segmentation. Setzer et al. employed deep-learning segmentation (DLS) utilizing the U-Net architecture on CBCT images comprising 61 lesioned and nonlesional roots to assess lesion detection accuracy, achieving excellent ratings for sensitivity (0.93), specificity (0.88), positive predictive value (0.87), and negative predictive value (0.93) [38]. In contrast, Orhan et al. employed a U-Net-like architecture for the detection of periapical lesions in CBCT images, utilizing binary voxel-based intersection and prediction metrics based on the true mask combination (IoU) to assess the model’s efficacy in predicting lesion localization. This deep learning model identified 142 out of 153 periapical lesions, yielding a reliability of 92.8% [46].

The current study has some limitations, including its focus on segmenting MB2 canals exclusively in maxillary first molars that had not undergone endodontic treatment. Our model did not deal with artifacts from root canal fillings employed in endodontically treated teeth, where the MB2 canal is overlooked. Moreover, the full-size scans were excluded from our study; instead, the models were trained solely on cropped images to minimize computational complexity, as advised by the computer scientist, in addition to using scans obtained with standard protocol which inevitably impacted the accuracy and generalizability of the model’s performance. Furthermore, the images used in our study were not classified according to MB2 canal locations (such as orifice, mid-third or apical third) and were loaded for training the model in a mixed manner. It is more valuable to identify the MB2 canal at which level along the root before endodontic treatment, however, this can be easily detected when segmentation is applied.

Future prospective studies are recommended to increase model accuracy in the determination of root canal type according to the most common classifications. Additionally, expanding the scope to include maxillary second molars, which exhibit a higher prevalence of MB2 canals, would provide a more comprehensive evaluation and improve the clinical applicability of the findings. Furthermore, Future studies could benefit from incorporating scans obtained through varied imaging protocols to enhance applicability across diverse clinical settings.

According to the results of the present study, we can conclude that the MB2 canal of maxillary first molars can be detected and segmented in CBCT images using CNN- and UNet-based artificial intelligence systems used in our study with high accuracy; however, the CNN model’s actual discriminative ability on new data is limited in the absence of segmentation. While the models show potential, further refinement and larger, higher-quality datasets are needed to improve their diagnostic reliability.

## Data Availability

The datasets used and/or analyzed during the current study are available from the corresponding author on reasonable request.
